# An international, open-access dataset of dental wear patterns and associated broad age classes in archaeological cattle mandibles

**DOI:** 10.1038/s41597-024-03377-y

**Published:** 2024-07-02

**Authors:** Eden Hill, Umberto Albarella

**Affiliations:** 1 Max Planck Institute for Evolutionary Anthropology, Leipzig, Germany; 2 Eberhard Karls University of Tübingen, Tübingen, Germany; 3 University of Sheffield, Sheffield, UK

**Keywords:** Databases, Animal physiology

## Abstract

Zooarchaeologists investigate past interactions between animals, humans, and their environments by analyzing the remains of archaeological fauna. Age-at-death distributions are fundamental to faunal analysis and are often estimated by comparing exposed dentine patterns to standardized tooth wear stages that have been associated with relative age classes. We present Bubona, an international dataset of dental wear patterns and associated broad age classes in archaeological cattle mandibles. Our open-access dataset of 1460 data entries from nine counties is being used to create tooth-type specific reference tables of probable age class attribution for cattle mandibles lacking complete dentition. Bubona is a valuable resource for the innovation of new systems of age estimation for cattle and it is the creators hope that researchers will continue to both help expand the dataset by contributing their own data, as well as utilize the data to refine and innovate age-at-death estimation methods.

## Background & Summary

Zooarchaeologists use a variety of methods to reconstruct profiles of animal assemblages from archaeological sites^1^. The age-at-death distributions of archaeological fauna can help to address many archaeological questions, ranging from hunting patterns to husbandry regimes, ritual behavior, cultural preferences etc. Their estimation is a critical aspect of zooarchaeological research^2,3^. The relative age-at-death of archaeological fauna is generally estimated through the examination of bones and teeth, which are commonly recovered and exhibit determinate growth^1–4^.

A fundamental zooarchaeological method which utilizes teeth to estimate the relative age-at-death of archaeological animal remains is the analysis of dental eruption and attrition^3^. Dental age in cattle may in turn be associated with broad or narrow chronological age classes (e.g.^5–12^). Bubona was created to synthesize Grant’s^6^ tooth wear stages with O’Connor’s^12^ broad age classes (Table 1) in an open-access dataset, in order to facilitate the age class attribution of cattle mandibles lacking complete posterior dentition. An additional age class, *Neonatal*, has been adapted into O’Conner’s original criteria^12^.

**Table 1:** O’Connor’s broad age classes (1988).

Age Class	Criteria
Neonatal	Fourth deciduous premolar not in wear
Juvenile	Fourth deciduous premolar in wear; first permanent molar not yet in wear
Immature	First permanent molar in wear; second permanent molar not yet in wear
Subadult	Second permanent molar in wear; third permanent molar not yet in wear
Adult	Third permanent molar in wear but not heavily worn; wear stage a - i*
Elderly	Third permanent molar heavily worn; wear stage j or beyond*

*Wear stages as defined by^6^.

Age-at-death frequency distributions, known as mortality profiles, derived from assemblages of wild animals (such as aurochs) can be utilized to extrapolate hunting tactics, seasonal killing, and seasonal occupation^3,13–15^. The mortality profiles of assemblages containing domesticated animals (such as cattle) may be interpreted to infer herd specialization and livestock management practices^10,16–21^. The value of age-at-death data cannot be understated and the authors are in the process of using Bubona to create and publish reference tables which correlate the development of the fourth premolars and molars with broad age classes (publication forthcoming). We strongly encourage researchers to submit their own data to be incorporated into Bubona, especially welcome data from archaeological contexts outside of the United Kingdom. Any submitted data would be vetted by the authors before inclusion into our dataset. We also whole-heartedly welcome other researchers to use our dataset to develop additional age-at-death estimation methods, or refine existing ones.

## Methods

Bubona is a collation of 1460 tooth eruption and wear data derived from both prerecorded and independently examined cattle mandibles housed in university collections and recovered from archaeological sites and across the world. The details of our data selection, collection, and compilation are described below.

### Data selection

Two criteria were observed in the selection of archaeological cattle and aurochs mandibles; (i) the presence of at least two molars or fourth premolars and (ii) the presence of erupted third molars, or the furthest back erupted molar if the third molar had not yet erupted.

**Fig. 1:**
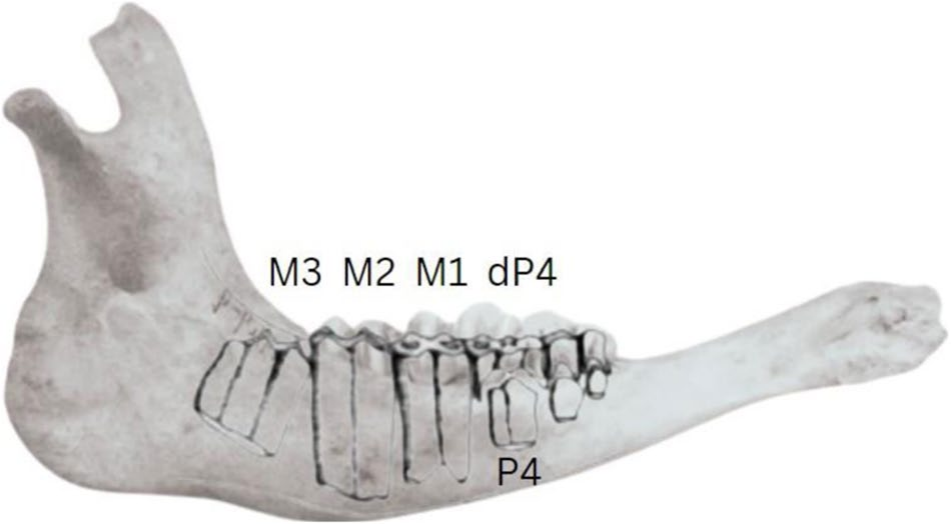
Dental schema of a cattle mandible adapted from^1^.

The presence of at least two molars or fourth premolars is a useful criterion because^12^ system of age classification is dependent upon the eruption and wear of molars/fourth premolars in association with one another. These teeth are abbreviated as dP4, P4, M1, M3, and M3 (Fig. 1). The examination of mandibles with at least two recordable teeth also reveals incidence of tooth wear stages which do not form parameters for age classes; e.g. it is the recording of tooth wear stages of the remaining molars and fourth premolar of an elderly mandible which reveals which non-M3 tooth stages are frequently exhibited by elderly individuals.

The direct attribution of the cattle mandibles to O’Connor’s broad age classes^12^ as opposed to Grant’s mandibular wear stages^6^ is possible only in mandibles which have the furthest back erupted molar present. Accordingly, only mandibles with erupted third molars or the furthest back erupted molar if the third molar had not yet erupted were incorporated into Bubona.

### Data collection

Pre-recorded data was included in our personally owned data^22–26^, and provided by Veronica Aniceti^27,28^, George Kazantzis^29,30^, Mauro Rizzetto^31–43^, Sofia Tecce (unpublished data from Rectory Farm, Lincolnshire, UK), Angela Trentacoste^44,45^, and Lizzie Wright^46–48^. Additional data was pulled from published sources^11,49–57^.

**Fig. 2:**
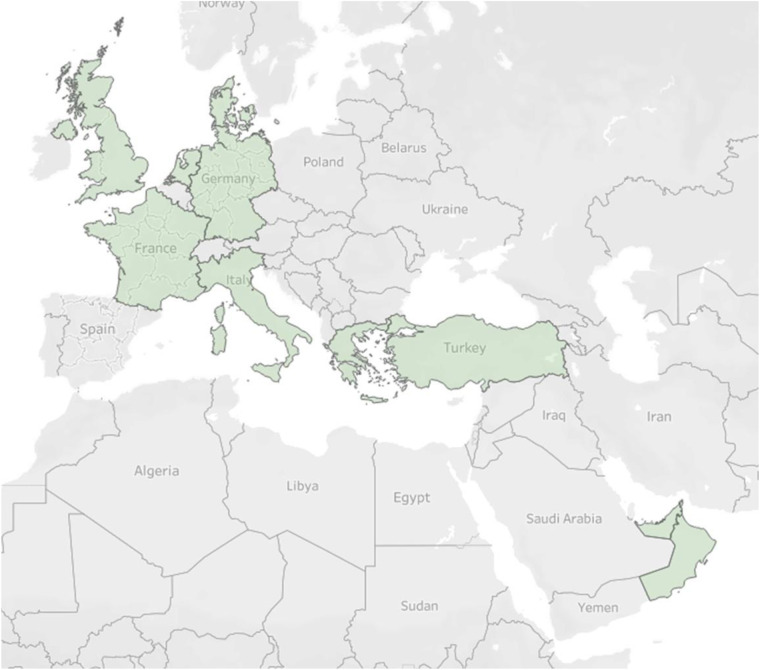
Map of countries represented in Bubona.

Independently recorded cattle mandibles were selected from multiple sites across France, Germany, and the UK (Chaplin Treasury, no known publication; Dangstetten Roman Camp^58^; Gurness^59,60^; Inveresk Gate^61^; Jarlshof^62^; Lazenay^63^; Links of Noltland^64^; Newstead^65^; Perth High Street^66^; Skeleton Green^67^) and analyzed by one of us (EH) in accordance with Grant^6^ and O’Connor^12^. Modern cattle mandibles from Germany, Oman, Turkey, and the UAE were also selected from the Tübingen University zooarchaeological collections. In total, cattle mandibles from 9 countries have been incorporated into Bubona (Fig. 2).

Access to the assemblage from the Lazaney site was facilitated by Robin Bendry and the University of Edinburgh, where the materials were being housed at the time of data collection. The assemblage from Skeleton Green was examined at the University of Sheffield (on loan from Hertford Museum). The remaining assemblages were examined at the National Museum Stores in Edinburgh under the supervision of Zena Timmons by permission of Jerry Herman and the National Museum of Scotland. The assemblage from Dangstetten Roman Camp, and all modern mandibles, were examined at the University of Tübingen zooarchaeological collections under the supervision of Angel Blanco-Lapaz by permission of Britt Starkovich.

### Data entry

Data recorded from all mandibles selected was entered into an Excel spreadsheet organized by

(i) archaeological site or modern context (see ‘Data Collection’), (ii) chronological context (iii) tooth wear stages^6^ recorded for each mandibular tooth (dP4, P4, M1, M2, M3), and

(iv) age classes (^12^, with the added class on *Neonatal*) recorded for each mandible. C represents crypt, V represents visible, H represents half-erupted, E represents erupted, and U represents unworn. The age class *Neonate* has been added to O’Connor’s age classes and is assigned to mandibles with an unworn dP4 (Table 1).

Reference publications and DOIs of faunal and archaeological reports and datasets were also included for each entry whenever possible.

### Taxa inclusion

Bubona is composed of tooth wear stage and age class data recorded for archaeological and modern domesticated cattle (*Bos taurus*) and aurochs (*Bos primigenius*).

## Data Records

The dataset is freely available as an .xlsx file on Zenodo^68^. Bubona includes 1460 data entries for cattle and aurochs mandibles recovered from 79 archaeological sites and 5 modern contexts. All entries have been associated with (i) an archaeological site or modern context, (ii) a chronological context (iii) tooth wear stages^6^ recorded for each mandibular tooth (dP4, P4, M1, M2, M3), and (iv) age classes^12^ recorded for each mandible.

## Technical Validation

Bubona is composed of lower posterior (dP4/P4, M1, M2, M3) tooth wear stages and broad age classes recorded in cattle and aurochs mandibles exhibiting the presence of at least two molars or fourth premolars as well as the presence of erupted third molars (or the furthest back erupted molar if the third molar had not yet erupted). All mandibles have been assigned a unique Bubona ID that is clearly associated with their original ID and all tooth wear stages and age classes can be checked either against their original datasets or the original data. In the case of original data, occlusal and buccal/lingual photographs taken of the mandibles that were selected for inclusion in Bubona and independently recorded in either the University of Sheffield, the University of Edinburgh, or the National Museum Stores in Edinburgh in 2018 or the University of Tübingen in 2023 are available for review on Zenodo^55,68^.

## Data Availability

No custom code has been used in the creation of the Bubona Dataset.

## References

[1] Davis, S. J. *The archaeology of animals*. Routledge (2012).

[2] Klein, R. G. & Cruz-Uribe, K. *The analysis of animal bones from archeological sites*. University of Chicago press (1984).

[3] Reitz, E. J. & Wing, E. S. (2008). *Zooarchaeology*. Cambridge University Press.

[4] Hillson, S. *Teeth*. Cambridge university press (2005).

[5] Grant. A. The animal bones. In: Cunliffe, B. (ed.), Excavations at Portchester Castle. Volume I; Roman. London: Society of Antiquaries, pp. 378–408 (1975).

[6] Grant, A. The Use of Tooth Wear as a Guide to the Age of Domestic Ungulates. In: B. Wilson, C. Grigson & S. Payne (eds.), Ageing and Sexing Animal Bones from Archaeological Sites (Oxford: BAR British Series 109), pp. 91–108 (1982).

[7] Grigson C (1982). Sex and age determination of some bones and teeth of domestic cattle: a review of the literature. Ageing and sexing animal bones from archaeological sites.

[8] Halstead, P. A study of mandibular teeth from Romano-British contexts at Maxey. In: Pryor, F. & French, C. (eds.), Archaeology and Environment in the Lower Well and Valley, Vol 1 (East Anglian Archaeology 27). Norwich: East Anglian Archaeology, pp. 219–224 (1985).

[9] Higham C (1967). Stock rearing as a cultural factor in Europe. Proceedings of the Prehistoric Society.

[10] Jones G, Sadler P (2012). Age-at-death in cattle: methods, older cattle and known-age reference material. Environmental Archaeology.

[11] Legge, J. Excavations at Grimes Graves, Norfolk 1972–76. Fascicule 4: Animals, Environment and Economy. British Museum Press: London (1992).

[12] O’Connor, T. Bones from the General Accident Site, Tanner Row. The Archaeology of York 15/2. London: Council for British Archaeology (1988).

[13] Klein R (1982). Age (mortality) profiles as a means of distinguishing hunted species from scavenged ones in Stone Age archeological sites. Paleobiology.

[14] Stiner MC (1990). The use of mortality patterns in archaeological studies of hominid predatory adaptations. Journal of anthropological archaeology.

[15] Zhang S, Li Z, Zhang Y, Gao X (2009). Mortality profiles of the large herbivores from the Lingjing Xuchang Man Site, Henan Province and the early emergence of the modern human behaviors in East Asia. Chinese Science Bulletin.

[16] Gillis R (2013). Sophisticated cattle dairy husbandry at Borduşani-Popină (Romania, fifth millennium BC): the evidence from complementary analysis of mortality profiles and stable isotopes. World Archaeology.

[17] Hesse, B. Slaughter patterns and domestication: the beginnings of pastoralism in western Iran. *Man*, 403–417 (1982).

[18] Kamjan S, Erdil P, Hummel E, Çilingiroğlu Ç, Çakırlar C (2022). Traction in Neolithic Çatalhöyük? Palaeopathological analysis of cattle and aurochs remains from the East and West Mounds. Journal of Anthropological Archaeology.

[19] Marciniak, A. The Secondary Products Revolution, mortality profiles, and practice of zooarchaeology. *Animal secondary products. Domestic animal exploitation in prehistoric Europe, the Near East and the Far East*, 186–205 (2014).

[20] Payne S (1973). Kill-Off Patterns in Sheep and Goats: The Mandibles of Asvan-Kale. Anatolian Studies.

[21] Reid A (1996). Cattle herds and the redistribution of cattle resources. World Archaeology.

[22] Albarella, U. *et al*. *Norwich Castle: Excavations and historical surveys 1987–98. Part III: a Zooarchaeological Study* (Vol. 22). Norfolk (2009).

[23] Albarella, U. & Davis, S. J. Mammals and birds from Launceston Castle, Cornwall: decline in status and the rise of agriculture. *Circaea*, **12**(1) (1996).

[24] Albarella, U. & Davis, S. J. The animal bones. In: A. Chapman (Ed.), West Cotton, Rounds: A Study of Medieval Settlement Dynamics AD 450–1450, Oxbow Books, Oxford, pp. 516–537 (2010).

[25] Albarella, U. *et al*. The animal bone. In *West Cotton, Raunds: A Study of Medieval Settlement Dynamics AD* 45*0–1450. Excavation of a deserted medieval hamlet in Northamptonshire, 1985–89* (pp. 516–537). Oxbow Books. (2010).

[26] Johnstone, C. & Albarella, U. The late Iron age and Romano-British mammal and bird bone assemblage from Elms farm, Heybridge, essex. *Internet Archaeology*, 40 (2015).

[27] Aniceti, V. *Animals and their roles in the medieval society of Sicily: from Byzantines to Arabs and from Arabs to Norman/Swabians* (Doctoral dissertation, University of Sheffield) (2019).

[28] Stallibrass S (1985). Some effects of preservational biases on interpretations of animal bones. *Palaeoenvironmental investigations: research design, methods and data analysis*. Oxford: British Archaeological Reports, International Series.

[29] Kazantzis, G. Animal bones from the Bronze Age pit-site of Kryopigado in Aliakmon of Voio. Report for the Ephorate of Antiquities of Kozani, Archaeological Museum of Aeani (2015).

[30] Kazantzis, G. *The Zooarchaeology of the Late Neolithic Strymon River Valley: The Case of the Greek Sector of Promachon-Topolnica in Macedonia, Greece*. BAR Publishing (2018).

[31] Beech, M. *Animal bones*. Pakenham (PKM 005), Suffolk. Unpublished zooarchaeological report, Suffolk County Council Archaeological Service (1991).

[32] Crabtree, P. J. *West Stow, Suffolk: Early Anglo-Saxon Animal Husbandry* (No. 47). Suffolk County Planning Department (1989).

[33] Crabtree, P. Zooarchaeology and colonialism in Roman Britain: evidence from Icklingham. Anthropological Approaches to Zooarchaeology: Colonialism, Complexity, and Animal Transformations. 190–194 (2010).

[34] Done, G. Animal Bone from Anglo Saxon Contexts. In: H. Hamerow, ed. Excavations at Mucking, Vol. 2: The Anglo Saxon Settlement (English Heritage Archaeological Report 21). London: English Heritage & British Museum Press, pp. 74–79 (1993).

[35] Hamilton-Dyer, S. Animal bone. In: Excavation of Neolithic, Late Bronze Age, Early Iron Age and Early Saxon features at St. Helen’s Avenue, Benson, Oxfordshire, J. Pine and S. Ford (eds), pp. 163-170. Oxoniensia 68: 131-178 (2003).

[36] Hamilton-Dryer S (2009). Animal bone (Web Report 11). Cambourne New Settlement. Iron Age and Romano-British settlement on the clay uplands of west Cambridgeshire.

[37] Ingrem, C. Animal bone. In Excavations at Oxford Science Park, Littlemore, Oxford, J. Moore (ed.), pp. 202–212. Oxoniensia 66: 163-219 (2001).

[38] Maltby, M. Animal bone. In The Roman and Early Anglo-Saxon settlement at Wantage, Oxfordshire. Excavations at Mill Street, 1993-4, N. Holbrook & A. Thomas (eds), pp. 155–163. Oxoniensia 61: 109–179 (1997).

[39] Maltby, M. Animal bone. In A Romano-British settlement to the rear of Denchworth Road, Wantage, Oxfordshire: evaluation and excavation in 1996 and 1998, N. Holbrook & A. Thomas (eds), pp. 320–325. Oxoniensia 66: 289–336 (2001).

[40] Powell, A. Clark K., & Serjeantson, D. The animal bones. In Asthall, Oxfordshire: excavations in a Roman ‘small town’, Thames Valley Landscapes Monograph No. 9, P. Booth (ed.), pp. 141–147. Oxford: Oxford Archaeological Unit (1997).

[41] Prummel W, Esser E, Zeiler JT (2013). The animals on the terp at Wijnaldum-Tjitsma (The Netherlands)–reflections on the landscape, economy and social status. Settlement and Coastal Research in the Southern North Sea Region.

[42] Rizzetto, M. *Developments in animal husbandry between the Late Roman period and the Early Middle Ages: a comparative study of the evidence from Britain and the Lower Rhineland* (Doctoral dissertation, University of Sheffield) (2019).

[43] Roberts, A. Animal bone. In: Excavations at Station Road, Gamlingay, Cambridgeshire, J. Murray and T. McDonald (eds), pp. 246–248. Anglo-Saxon Studies in Archaeology and History 13: 173-330 (2006).

[44] George D (2017). Recent Research in Cavità 254 (Orvieto, Italy). Etruscan Studies.

[45] Trentacoste, A. C. *The Etruscans and their Animals: The Zooarchaeology of Forcello di Bagnolo San Vito (Mantova)* (Doctoral dissertation, University of Sheffield) (2014).

[46] Wright, E., Corbino, C., Albarella, U. The animal bones from Norton Priory, Runcorn, Cheshire. Unpublished report (2016).

[47] Wright L (2016). Open Context..

[48] Wright E, Tecce S, Albarella U (2019). The use of animals at Roman roadside settlements in Britain: contextualizing some new results from Ware, Hertfordshire. Oxford Journal of Archaeology.

[49] Davis, G. W. *The fate of neonate calves. A discussion of the bovine infant health implications of dairying in antiquity, using archaeozoological studies of six Orcadian contexts* (Doctoral dissertation, University of Bradford) (2011).

[50] Gidney, L. Leicester, The Shires, 1988 Excavations: the animal bones from the Medieval Deposits at St. Peter’s Lane. Ancient Monuments Laboratory Report 116/91. London (1991a).

[51] Gidney, L. J. Leicester, The Shires, 1988 Excavations: the animal bones from the medieval deposits at Little Lane. Ancient Monuments Laboratory Report 57/91. London (1991b).

[52] Orton DC, Russell N, Twiss K, Martin L, Frame S (2013). Open Context.

[53] Serjeantson, D. Review of animal remains from the Neolithic and Early Bronze Age of Southern Britain (2011).

[54] Albarella, U. & Johnstone, C. The early to late Saxon animal bones excavated in 1995 from Kings Meadow Lane, Higham Ferrers, Northamptonshire. *Ancient Monument Laboratory Report* **79** (2000).

[55] Hill E (2023). Zenodo.

[56] Pine J, Ford S (2003). Excavation of Neolithic, Late Bronze Age, Early Iron Age and Early Saxon Features at St. Helen’s Avenue, Benson, Oxfordshire. Oxoniensia.

[57] Thomas, R. *Animals, economy and status: integrating zooarchaeological and historical data in the study of Dudley castle, West Midlands (c. 1100–1750)*. BAR Publishing (2005).

[58] Fingerlin G (1971). Rheinheim - Dangstetten. A legionary camp from early Roman times. Archaeological News from Baden.

[59] Hedges, J. Bu, Gurness and the Brochs of Orkney. Oxford: British Archaeological Reports, pp. 637 (1987).

[60] Schofield, J. *et al*. Excavations south of Edinburgh High Street, 1973-4. In *Proceedings of the Society of Antiquaries of Scotland* (Vol. 107, pp. 155–241) (1978).

[61] Bishop M (2004). Inveresk Gate: Excavations in the Roman Civil Settlement at Inveresk, East Lothian, 1996–2000. Star Monograph.

[62] Platt, M. Report on the Animal Bones. In: J. Hamilton (ed.), Excavations at Jarlshof, Shetland: Ministry of Works Archaeological Reports No. 1. Edinburgh: Her Majesty’s Stationery Office, pp. 212–215 (1956).

[63] Buchsenschutz, O. & Ralston, I. L’occupation de l’âge du Fer dans la vallée de l’Auron a Bourges. Installations agricoles, funéraires et cultuelles (Xe – ler siecle avant J.-C.) Monographie 2001- 2002. Bourges/Tours: Ville de Bourges, pp. 1–222 (2001).

[64] Clarke, D. Excavation of the Neolithic Settlement at the Links of Noltland, Westray, Orkney 1978–1981. Part One: The History and Discovery of the Site, the Excavation Strategy and a Summary of the Results. Unpublished report prepared for the Scottish Development Department (1991).

[65] Ewart, J. Animal Remains. In: J. Curle (ed.), A Roman Frontier Post and its People. The Fort at Newstead. Glasgow: Glasgow University Press, pp. 362–377 (1911).

[66] Hodgson, G. *et al*. Perth High Street Archaeological Excavation 1975–1977: Fascicule 4. Living and Working in a Scottish Medieval Burgh. Environmental Remains and Miscellaneous Finds. Perth: Tayside and Fife Archaeological Committee, pp. 161 (2011).

[67] Partridge, C. Skeleton Green: A Late Iron Age and Romano-British Site. Society for the Promotion of Roman Studies, pp. 1–359 (2012).

[68] Hill E, Albarella U (2023). Zenodo.

